# Country economic status is strongly associated with burn survival - validation of the (modified) ABSI

**DOI:** 10.1186/s12939-024-02353-7

**Published:** 2025-01-09

**Authors:** Julia Elrod, Christoph Mohr, Ludvik Branski, Joshua M. Peterson, Fionna M. Wood, Dale W. Edgar, Pius Agbenorku, Shobha Chamania, Anant Sharma, Flavio N. Novaes, Jean Bosco Katabogama, Michael Boettcher, Konrad Reinshagen, Ingo Koenigs

**Affiliations:** 1 Department of Pediatric Surgery, University Medical Center Hamburg-Eppendorf, Martinistrasse 52, 20246 Hamburg, Germany; 2 Department of Pediatric Surgery, University Medical Center Mannheim, Heidelberg University, Theodor-Kutzer-Ufer 1-3, 68167 Mannheim, Germany; 3 Department of Surgery, University of Texas Medical Branch, 301 University Blvd, Galveston, TX 77555-0527 USA; 4 Burn Injury Research Unit (BIRU), University of Western Australia, 35 Stirling Hwy, Perth, WA 6009 Australia; 5 Institute for Health Research, The University of Notre Dame Australia, Fremantle, 6160 Australia; 6 Safety and Quality Unit, Armadale Kalamunda Group Health Service, East Metropolitan Health Service, Mt Nasura, 6112 Australia; 7 Department of Surgery, School of Medical Sciences, Kwame Nkrumah University of Science and Technology, Kumasi, Ghana; 8 Choithram Hospital and Research Centre, Indore, Madhya Pradesh India; 9 Irmandade de Misericordia de Campinas, Av. Julio de Mesquita, 571 Cambini/Campinas-JP, São Paulo, CEP:13024-180 Brazil; 10 Ruhengeri Referral Hospital, P.O.Box 57, Musanze District, Musanze, Northern Province Rwanda; 11 Department of Pediatric Surgery, Altona Children’s Hospital, Bleickenallee 38, 22763 Hamburg, Germany

**Keywords:** Burns, Outcome, ABSI, HDI, Burn survival, International burn care, Health inequalities, Country economic status

## Abstract

**Background:**

Predicting burn-related mortality is vital for family counseling, triage, and resource allocation. Several of the burn-specific mortality prediction scores have been developed, including the Abbreviated Burn Severity Index (ABSI) in 1982. However, these scores are not tested for accuracy to support contemporary estimates of the global burden of burn injury. This study compares burn mortality across countries with varying economic levels, as indicated by the Human Development Index (HDI), to assess if a modified ABSI (mABSI) offers enhanced predictive accuracy.

**Methods:**

A retrospective study was performed, including over 90,000 patients from seven sources in five continents, including two burn registries and five referral burn centers. Data from 2015 to 2019 were collected, including age, gender, presence of inhalation injury, full-thickness burn, percentage of total body surface area, and outcome. The participating countries were classified based on the HDI and mortality was predicted using both the original and the modified ABSI.

**Results:**

After removal of incomplete data, 74,460 sets remained for the analysis. Significant variations in population demographics, sample sizes, total body surface area (TBSA), and age distributions across the studied regions were noted. The modified ABSI demonstrated a smaller deviation from the 95% CI of the true survival probability than the original ABSI in countries with a very high or high HDI, indicating improved accuracy here. In these countries, the original ABSI overestimates mortality. Conversely, in countries with a middle or low HDI, the original ABSI remains more accurate, reflecting the significantly greater burn related mortality in countries with a low HDI.

**Conclusion:**

In burn patients, the probability of survival remains highly dependent on the level of development of a country. Our results support the use of the modified ABSI in countries with a (very) high HDI, yet in resource constraint settings, the original ABSI seems to provide more accurate predictions. These findings underscore the need for adaptive models that take into account socioeconomic variables, potentially guiding health policy adjustments and emphasizing the necessity of resource allocation and training in lower HDI countries. Such adaptations could enhance clinical outcomes and reduce disparities in burn care effectiveness globally.

**Supplementary Information:**

The online version contains supplementary material available at 10.1186/s12939-024-02353-7.

## Background

Predicting burn-related mortality is not only important for family counseling and decision-making support, triage purposes, and capacity planning, but is also advantageous for scientific purposes, e.g., for systematic assessment of treatment modalities and for comparison of international standards of burn care [1–4].

Several burn specific formulae have been developed over the last decades, such as the Abbreviated Burn Severity Index (ABSI) [5] the classic and the revised Baux score [6] and the Ryan scorer [7], of which the ABSI is used most commonly. Since its introduction in 1982 both intensive care and burn surgery have made considerable progress, leading to a decrease in burn related mortality, especially in high income countries (HICs) [8, 9]. The score has been validated multiple times in the past, but the question whether the ABSI still predicts mortality accurately has been raised by several authors. They have suggested updating the ABSI to reflect improvements in burns treatment or alterations in demographic data and risk factors in the past decades [8, 10–12].

The research gap identified in this study addresses the untested accuracy of burn mortality prediction across countries with varying Human Development Indexes (HDIs), highlighting a potential limitation as most mortality prediction scores, including the ABSI, were developed in high-resource settings such as the USA [13]. This discrepancy raises concerns about the applicability of existing models in countries with lower resources, such as intensive care units, skilled manpower and costly instruments or dressing materials, where treatment availability and quality can vary significantly [14, 15]. In high-income nations, significant progress in the fields of prevention, management, and rehabilitation has led to a substantial reduction in the incidence, severity, mortality, and disability related to burns [16, 17]. In contrast, low- and middle-income countries (LMICs), continue to face a high burden of burns. The discrepancy in outcomes between these regions is exacerbated by varying levels of access to advanced medical infrastructure and trained healthcare professionals [18–20]. Many studies focus on the cause of burns, instead of on the global burden, which however is important for the development of intervention strategies [21].

In a recent study with almost 15,000 patients from the German Burn Registry from 2014 to 2018 we demonstrated the ABSI to overestimate mortality in severely burned patients and introduced a modified version of the ABSI, referred to as the mABSI [22]. In particular, this modified score excluded sex as a predictive parameter and applied a new age point scale. The question arose, whether this observation is valid globally or limited to burns patients from highly developed countries.

The objective of this study is to compare burn mortality globally in the context of distinct HDI levels and to evaluate whether the modified ABSI (mABSI) provides more accurate predictions of survival probabilities. By addressing these gaps, this study contributes significantly to the literature, providing a comparative analysis of burn mortality across different economic and developmental contexts and highlighting the need for adaptable and region-specific mortality prediction models. Over 74,000 patients from 7 countries were included in this analysis, providing a large dataset for evaluating the effectiveness of the modified index.

## Methods

### Study design

A retrospective study was performed as a joint international project, including adult and pediatric burn patients from 2 burn registries and 5 referral burn centers from 5 continents.

### Data collection

Data from consecutive admissions to each of these 7 sources from 2015 to 2019 were collected from patients with burns, whereby the exact period of data collection differs slightly from center to center. Furthermore, the size and representativeness of the datasets collected varied significantly between the centers, which partly significant differences regarding TBSA and age of the patients, see supplementary material. In HIC countries data was generally extracted from registries, whereas in LMIC it was generally based on manual and retrospective reviews of handwritten medical records due to the absence of robust, electronic healthcare information systems in these regions, posing challenges to systematic data gathering and validation. The following demographic and burn-related data were collected: age, gender, presence of inhalation injury, presence of full thickness burn and percentage of total body surface area, outcome (death or survival).

The participating data sources were:

1. The burn registry of the American Burns Association [23] (USA).
2. International burn registry of German Speaking countries (Austria (AT), Switzerland (CH), Germany (DE)) from the German Society for Burn Treatment (DGV) [24] henceforth referred to as AT/CH/DE.
3. State Adult Burn Unit, Fiona Stanley Hospital, Western Australia [25] (AU).
4. Burns Unit of Komfo Anokye Teaching Hospital, School of Medical Sciences, Kwame Nkrumah University of Science and Technology, Kumasi, Ghana [26] (GH).
5. Burn Unit of Choithram Hospital, Research Center in Indore, India [27] (IN).
6. Burn Center, Irmandade de Misericordia de Campinas, Brazil [28] (BR).
7. Ruhengeri Referral Hospital, Musanze District, Northern Province, Rwanda [29] (RW).

Data quality was ensured by removing inconclusive or missing data sets. Standard abbreviations were used for countries, as ISO 3166-1, alpha 2 code [30].

### Human development index

Countries were classified according to the Human Development Index. The HDI is a composite index, measuring the average achievements in key dimensions of human development: life expectancy, education and standard of living. It is calculated on the basis of life expectancy at birth, the average number of years of schooling for adults and the expected duration of schooling for children, and gross national income per capita adjusted for purchasing power. Income is included in the calculation logarithmically in order to consider the decreasing importance of income in high areas.

Countries are grouped into four categories based on fixed cutoff points: A HDI < 0.550 is defined as low development (category D), a HDI of 0.550–0.699 as a medium human development (category C), a HDI of 0.700–0.799 as a high human development (category B), and HDI ≥ 0.800 (category A) as very high human development [13].

### Analysis of mortality and comparative validation of the ABSI and mABSI

The ABSI is a tool to specify the probability of survival in burns using 3 trauma-specific (burned body surface, presence of inhalation injury, presence of full thickness injury) and 2 patient-specific (age, gender) parameters. The individual parameter scores add up to the Total Burn Score defining the estimated survivability. With the mABSI, gender is excluded as a non-relevant parameter and the point distribution for the present age is adapted according to the non-linear age distribution of mortality. ABSI and mABSI with their different parameters are shown in Table 1.

**Table 1:** Definition of ABSI [5] and mABSI [22], probability of survival of the different total burn score groups

	ABSI (original)		Modified ABSI	
Variable	Patient characteristics	Score	Patient characteristics	Score
**Sex**	Female	1		
	Male	0		
**Age**	0–20	1	0–40	1
	21–40	2	41–70	2
	41–60	3	71–80	3
	61–80	4	81–90	4
	81–100	5	91–100	5
**Inhalation injury**		1		1
**Full thickness burn**		1		1
**Total body surface area burned (TBSA, %)**	1–10	1	1–10	1
	11–20	2	11–20	2
	21–30	3	21–30	3
	31–40	4	31–40	4
	41–50	5	41–50	5
	51–60	6	51–60	6
	61–70	7	61–70	7
	71–80	8	71–80	8
	81–90	9	81–90	9
	91–100	10	91–100	10
**Total Burn Score**	**Threat to life**	**Probability of survival**		
2–3	Very low	≥ 99%		
4–5	Moderate	98%		
6–7	Moderately severe	80–90%		
8–9	Serious	50–70%		
10–11	Severe	20–40%		
12–13	Maximum	≤ 10%		

Each patient was scored using the original and the modified ABSI (mABSI) and was categorized according to the ABSI groups (2–3, 4–5, 6–7, 8–9, 10–11, > 12). Actual (true) probability of Survival and 95% CI were calculated for each group.

To calculate how accurately the ABSI and the mABSI describe the real data, the deviation of the mean of the ABSI from the 95% CI of the true survival was determined. If the 95% CI and the range of the ABSI intersect, deviation was set to zero.

### Statistics

Statistical analysis was performed using the python library SciPy (Python Software Foundation) [31]. Means, standard deviations (SD) and confidence intervals (CI) were calculated and plotted in a descriptive manner. For each country, scatter plots for mortality were drawn and line plots for grouped ABSI and mABSI calculations were created. For the line plots, missing values or groups with less than 3 patients were omitted.

## Results

A total of 90,530 patient data sets were collected. After removal of incomplete data, 74,460 sets remained. Data from countries of all HDI groups were included: D – Rwanda; C – India, Ghana; B – Brazil; A – USA, Austria/Switzerland/Germany, Australia. Details concerning demographics and injury related data can be found in Table 2. Distribution of total body surface area (TBSA) and age differ significantly between the data sets from the different regions, details can be found in the supplement (*Supplementary material 1–7*). As shown, data from Australia and Brazil are from adult burn centers only.

**Table 2:** Overview of the patient datasets included in the study. Sources: USA, Austria (AT), Switzerland (CH), Germany (DE), Australia, Ghana, India, Brazil and Rwanda. Basic patient demographics and injury related data are indicated.

Source		*N*° of patients	Years	Pediatric patients included	Male Gender [%]	TBSA [mean ± SD]	Presence of IHT [%]	Presence of full thickness burn [%]
USA		54,335	2015–2018	yes	67.28	7.50 (11.98)	5.70	30.53
AT/CH/DE		14,782	2015–2018	yes	62.50	9.41 (13.11)	5.70	22.10
Australia		3,212	2009–2019	no	69.80	4.90 (7.91)	2.09	11.89
Ghana		772	2009–2019	yes	53.89	30.93 (20.98)	1.17	100.00
India		744	2014–2019	yes	55.11	33.09 (25.18)	41.26	76.34
Brazil		403	2016–2019	no	68.24	17.60 (17.02)	14.64	59.80
Rwanda		212	2015–2019	yes	56.60	14.51 (13.29)	4.25	6.60
Total		74,460	-	-	66.15	8.34 (12.28)	5.90	29.32

**Fig. 1:**
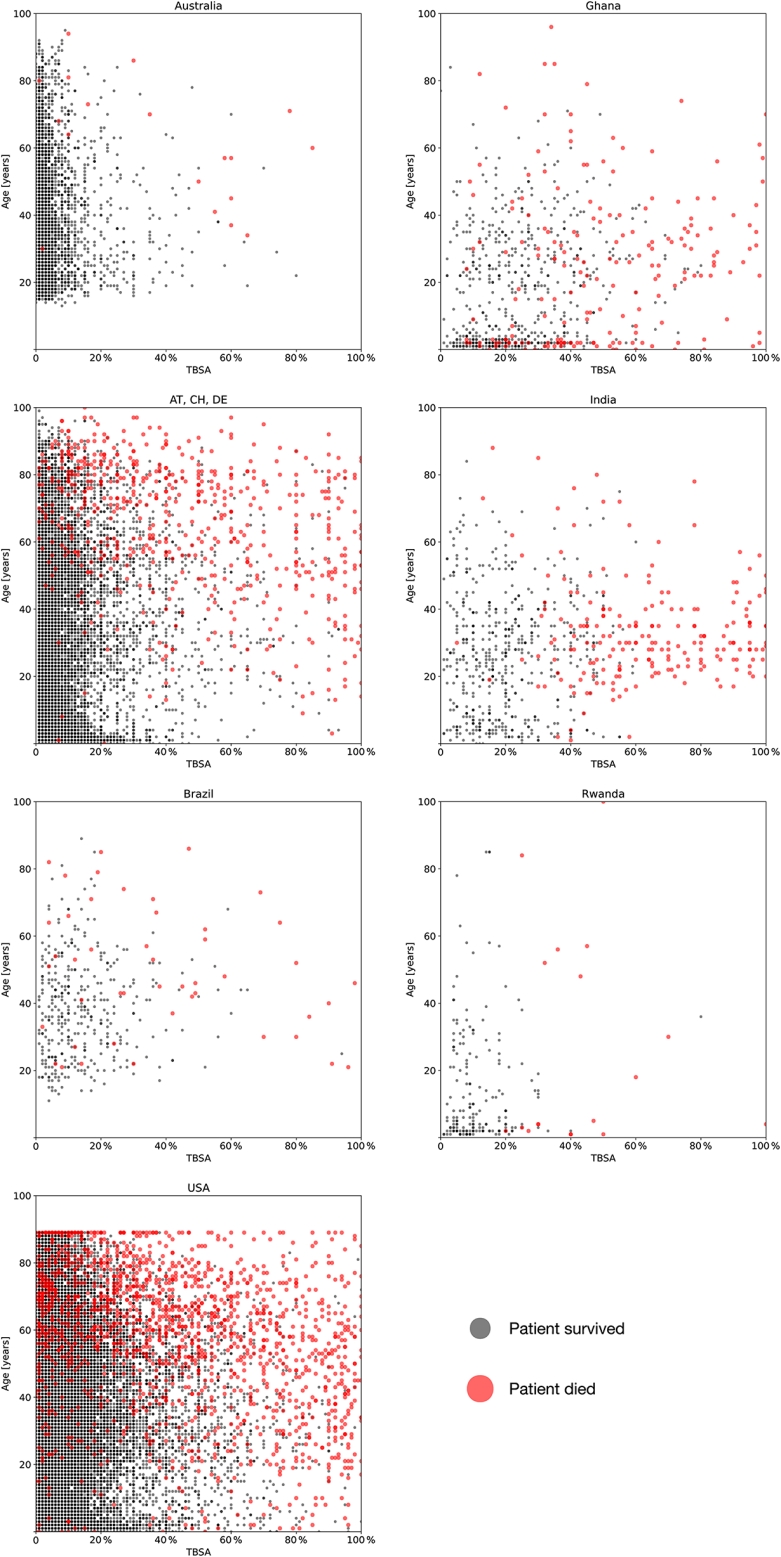
Burn related mortality as a function of TBSA and age. Each of the survivors (grey) and deceased (red) are illustrated for each of the countries included in the analysis. The shading of the point takes into account the fact that more than one patient falls on many points. Red data points are enlarged for clarity. Note that data from Australia and Brazil are from adult burn centers only, and only include few minor patients

In a first step, burn related mortality was displayed as a function of TBSA and age as to demonstrate the difference in survival patterns between the different countries, see Fig. 1.

**Fig. 2:**
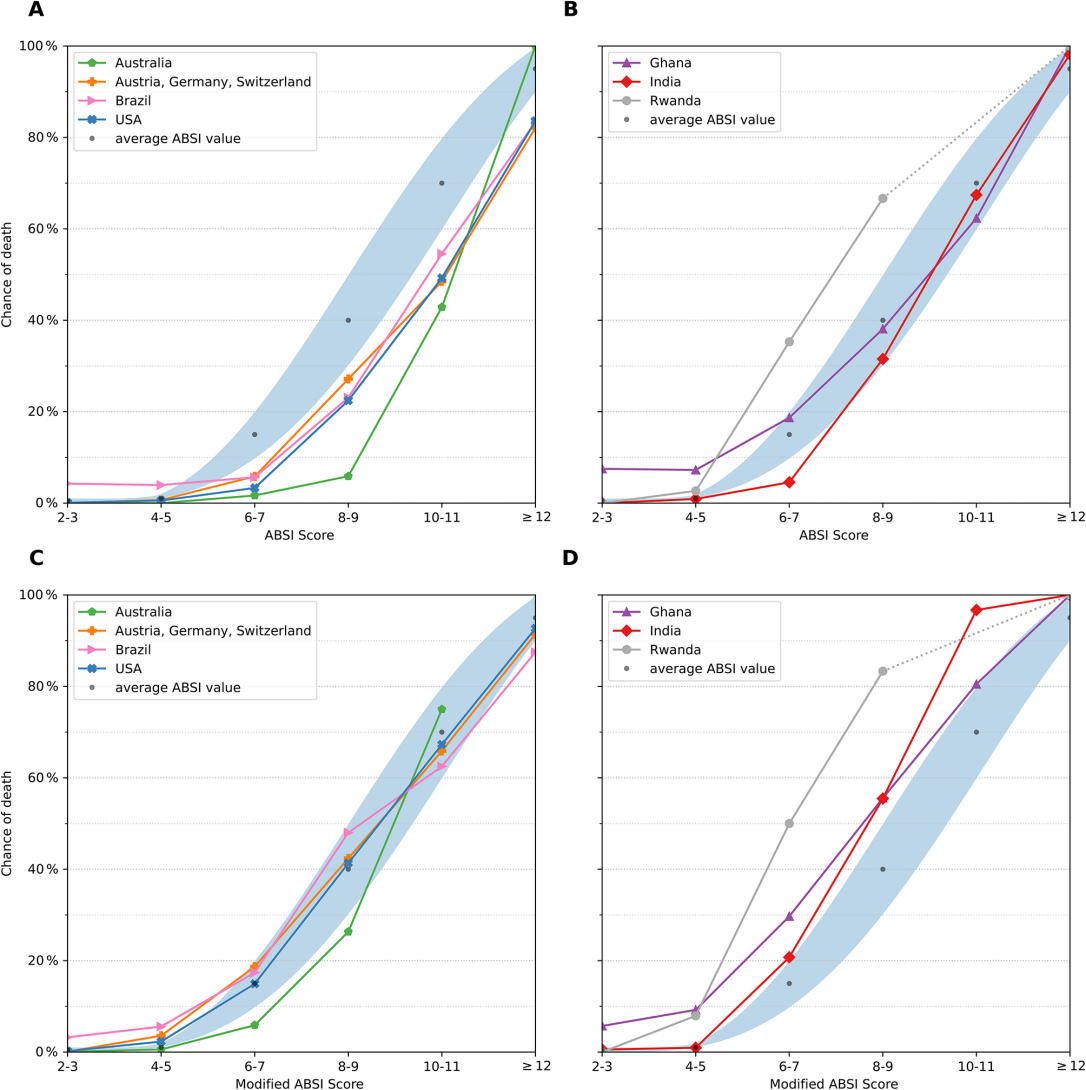
Line plots of the ABSI and the mABSI. Patients were scored using the (original) and the modified ABSI (mABSI) and were categorized according to the ABSI groups (2–3, 4–5, 6–7, 8–9, 10–11, > 12). Countries are grouped according to the HDI: **A**: ABSI of very high and high developed countries. **B**: ABSI of medium and low developed countries. **C**: mABSI of very high and high developed countries. **D**: mABSI of medium and low developed countries. The probability of death, as predicted by the ABSI is shown as a blue shaded area, the mean of this interval is depicted as black dots

Next, each patient was scored according to the original and the modified ABSI (mABSI), for the purpose of validation. Countries were grouped according to the HDI, see Fig. 2A-D.

Next, the accuracy of the ABSI and the mABSI to predict mortality was calculated, see Table 3. In brief, for countries with a very high to high HDI (USA, AT, CH, DE, AU, BR), the mABSI outperforms the accuracy of the ABSI, whereas in the remaining countries (GH, IN, RW) the original ABSI is superior in prediction of mortality.

**Table 3:** Accuracy of the two scoring systems (ABSI and mABSI). Calculations were performed for the data from the two burn registries (US and DE/ACH) and for each of the single countries (Brazil (BR), India (IN), Ghana (GH) and Rwanda (RW). Deviation of the mean of the ABSI and the mABSI from the 95% interval of the true survival was calculated separately for each of the defined ABSI scores. Mean deviation over the entire ABSI respective mABSI (deviation mean) is depicted in the column to the right. Here, for each country, the more accurate scoring system was marked in bold

Country	Scoring system	Deviation per Score						Deviation mean
		2–3	4–5	6–7	8–9	10–11	>= 12	
USA	ABSI	0.32	1.26	10.93	15.24	17.28	9.63	9.11
	**mABSI**	0.22	0.05	0.00	0.00	0.00	0.00	**0.04**
AT/CH/DE	ABSI	0.33	0.85	7.39	8.84	15.62	10.23	7.21
	**mABSI**	0.27	1.00	1.15	0.00	0.00	0.00	**0.40**
AU	ABSI	0.00	1.33	9.63	17.65	3.14	0.00	5.29
	**mABSI**	0.15	0.21	0.00	0.00	0.00	0.00	**0.06**
BR	ABSI	1.74	0.00	2.54	1.55	0.00	0.00	0.97
	**mABSI**	1.62	1.43	0.00	0.00	0.00	0.00	**0.51**
IN	**ABSI**	0.00	0.00	4.36	0.00	0.00	0.00	**0.73**
	mABSI	0.00	0.00	0.00	6.98	15.22	0.00	3.70
GH	**ABSI**	4.81	3.52	0.00	0.00	0.00	0.00	**1.39**
	mABSI	3.87	5.71	9.78	4.89	0.00	0.00	4.04
RW	**ABSI**	0.00	0.00	3.32	0.00	-	0.00	**0.66**
	mABSI	0.00	2.48	12.87	4.13	-	0.00	3.90

## Discussion

Global burn care lacks sufficient data on outcome, clinical and even epidemiological questions in general [32]. Data on burn related outcome are published almost exclusively from countries with a high HDI [33]. It is therefore difficult or almost impossible to carry out global analyses of the consequences of burns. The aim of the present study was to fill this gap: Its objective was to compare burn mortality globally in over 74,000 patients from 7 countries in the context of distinct human development indices. Moreover, we evaluated whether the original or the modified ABSI are more accurate in predicting the probability of survival.

This study showed that with the ABSI the following dualism emerged: For countries with a very high or high HDI, such as USA, AT/CH/DE, AU and BR the mABSI was more accurate than the original ABSI. In these countries, the use of the original ABSI leads to a significant overestimation of mortality. In contrast, the original ABSI remains more accurate in countries with a medium and low human development index such as GH, IN and RW. Here, the use of the mABSI leads to a significant underestimation of mortality, see Table 2; Fig. 2. The study thus highlights the importance of periodically updating and modifying scoring systems to reflect changes in burn care and demographics. As shown in Table 1 and the supplementary material, the demographic and injury data differ between countries. For example, there is a marked difference in the extent of injury between the lower HDI countries, which generally have a higher proportion of patients with higher TBSA, and the higher HDI countries, where major burns have become rare. Note that mortality as a function of TBSA and age also shows significantly different patterns between countries, see Fig. 1.

The findings regarding overestimation of burn related mortality in countries with a high or very high level of development, using the original ABSI are supported by previous publications [8] which is not surprising since significant progress has been made in burn care since the introduction of this score in 1982 in the USA [5, 9, 34]. On the contrary, the original ABSI, developed more than 40 years ago, still describes the probability of survival in investigated countries with low and medium HDI, despite significant development in burn care.

These results also indicate that, given the wide disparities in levels of development around the world, it is not possible to produce a globally applicable score to accurately predict survival. Instead, the data suggest that either a scoring system should be developed that takes into account the HDI or, alternatively, the dualism shown here should be followed: For highly developed countries (very high and high HDI) the mABSI can be used, for less developed countries (medium and low HDI) the ABSI. Of note, merely the outcome data from registries (US & AT/CH/DE) reflects nationwide data, whereas included data from the other countries only represents outcome from single burn centers and referral hospitals. Nevertheless, the data in this study are within the expected range, based on a review of the few available data from previous studies in these countries or comparable regions [35–39]. Thus, an approximation of national mortality rates based on these monocentric data - in the absence of national registries and the scarce availability of outcome data from less developed countries - appears to be a sufficiently accurate approach [33].

In high HDI countries, the proportion of small burns was higher than in the other countries in our study, which was also shown in previous studies with lower affected median TBSA in countries with a higher HDI [40]. Jacobs et al. also described a longer length of stay in HDI countries. Furthermore, similar to our results, this study shows lower mortality in these countries despite higher rates of associated injuries. Overall, with this in mind and the fact that only inpatients were analyzed, it must also be discussed that treatment strategies and reasons for admission will certainly differ between countries and are likely to be influenced by cost coverage and the health care system with different reimbursements [40]. A recent cross-sectional survey in LMICs also showed significant variation in the proportion of large burns, even between countries in the same HDI class. The proportion of burns with > 20% TBSA ranged from 19–81% [41]. As the influence of body surface area is an important parameter in determining the ABSI, it can be assumed that this particular inhomogeneity does not represent a significant bias in the results, because the increased body surface area affected will increase the ABSI accordingly [5, 22]. Over the past few decades, the validity of the ABSI as a prognostic tool for patients with burns of all sizes has been repeatedly demonstrated [42–46].

In general, countries with higher HDIs tend to have much higher per capita health expenditure and more doctors per 1000 inhabitants than countries with lower HDIs [47, 48].

The US spend 214 times more on healthcare per capita than Rwanda does [47], and gross domestic product (GDP) is 75 times higher [49]. Although health expenditure differs significantly even in countries with a very high HDI - e.g., per capita expenditure in the US is more than twice as high as in Germany or Australia - this does not lead to relevant differences in the probability of survival. The number of doctors per 1000 inhabitants varies widely across the world: for example, in Rwanda it is 0.1 and in India it is 0.7, while it is 4.4 for Switzerland and Germany likewise and even 5.3 for Austria [48]. There are also significant differences between the countries included in this study in terms of relative health expenditure: In the USA, 15.5% of GDP is spent on health, in Germany 10.6%, in Switzerland 10.5%, in Austria 9.8%, in Australia 9.8%, in Brazil 9.3%, in Rwanda 6.1%, in Ghana 3.4% and in India 3.0%.

While the World Bank’s data indicates that there are also significant differences in health spending and the number of doctors among the very high developed countries, our data do not suggest a clear difference regarding their ABSI scoring. Thus, beyond a certain level, money might not lead to pronounced improvement in survival rates. For detailed information see Table 4.

**Table 4:** Comparative compilations of health expenditure per capita, GDP per capita, physicians per 1000 people, population and HDI. Data retrieved from the World Bank and the United Nations

Country	Health expenditures per capita & year in US $ (2019) [47]	GDP per capita in US $ (2021) [49]	Physicians per 1000 people (2018/2019/2020) [48]	Population (2021) [50]	HDI 2021 [51]
US	10,921	70,248	2.6	331,893,740	0.921
AT/ CH/ DE	5,242 9,666 5,440	53,637 91,991 51,203	5.3 4.4 4.4	8,955,800 8,703,410 83,196,080	0.916 0.962 0.942
AU	5,427	55,100	4.1	25,688,080	0.951
BR	853	9,130	2.3	214,326,220	0.754
IN	63	2,120	0.7	1,407,563,840	0.633
GH	75	2,220	0.2	32,833,030	0.632
RW	51	830	0.1	13,461,890	0.534

The strong correlation between HDI and burn injury related survival shown in our analysis is consistent with the Global Burden of Disease (GBD) 2017 [52] and 2019 [32] studies. These studies analyze global epidemiology and trends in burns and highlight a general lack of epidemiological data from many low- and middle-income countries (LMIC). According to the GBD 2019 study, approximately 110,000 people die from burns each year. The vast majority of these are in low- and middle-income countries [32, 53]. Similar to our study, in a retrospective study of 189 countries, Peck et al. found a strong statistical association between death from thermal injuries and gross national income per capita (*R* = -0.36). The authors also demonstrated an association between the Gini coefficient and burn mortality (*R* = 0.17) by combining economic data from the World Bank and mortality data from the WHO [54]. The Gini coefficient is a statistical measure of the inequality of wealth distribution within a country.

Data from the included clinics in the different countries suggest that a lower HDI could be reflected by an additional point in the mABSI, where a higher score is correspondingly associated with a poorer prognosis. Adding one point per lower HDI class in our study cohort (B plus 1, C plus 2, D plus 3) would result in a comparable predictive value of the mABSI for all countries studied.

A general statement regarding an adaptation of the mABSI for the HDI classes is not possible due to the fact that only a few countries from the different classes were examined. If further investigations confirm this trend, a globally applicable version of the mABSI could be conceivable in the future, taking into account the HDI.

“Closing the gap in a generation” aimed by the WHO Commission on Social Determinants of Health in 2008 seems to be still far away to date, especially in global burn care [55]. A lot of work remains to fulfill the official theme of the 2012 *International Society for Burn Injuries* congress “One World, One Standard of Burn Care”.

### Limitations

This study has a number of limitations. First, it should be noted that the data are limited to patients from 7 regions/countries of the world. Thus, at most, the influence of the HDI on mortality and the accuracy of the ABSI can be shown by way of example. Larger studies from a global burn registry would be needed to remedy this. Furthermore, in some countries, only patients from a single hospital were included. This may have a non-negligible bias in patient population and mortality due to the different types of hospital, such as referral hospitals etc. Another weakness of the study is the very unevenly sized patient cohorts, which may affect the validity and reliability of our findings. The absence of robust, electronic healthcare information systems in less developed regions posed significant challenges to systematic data gathering and analysis. This situation also restricts the possibility for data validation, further affecting the representativeness and generalizability of the results. Accordingly, great care was taken to ensure that the participating centers carried out a complete, reliable and, above all, systematic survey of their patients in order to minimize possible bias.

These factors underscore the need for investment in healthcare infrastructure and data management systems in these settings to support better epidemiological surveillance and healthcare outcomes.

## Conclusion

For burn patients, the likelihood of survival depends strongly on the level of development of a country. In countries with a very high or high HDI, the modified ABSI is more accurate, as the original ABSI overestimates mortality. Conversely, in regions with lower HDIs, the original ABSI still provides a more accurate assessment, reflecting the greater challenges in managing burn injuries due to limited healthcare resources.

These results show that it is not possible to create a globally applicable score that accurately predicts survival. The study also highlights the importance of regularly updating and modifying scoring systems to reflect changes in burn care worldwide. Implementing such models could guide improvements in local health policies, particularly in enhancing burn care facilities and training for healthcare professionals in under-resourced areas. In addition, the study points out that a reduction in global burn mortality can only be achieved through a reduction in global poverty and provision of adequate and equitable health care for all.

Further research should explore the development of specific burn severity indices that better account for disparities in healthcare capabilities and socio-economic conditions. This would not only improve the management of burn injuries globally but also support broader efforts to equalize healthcare outcomes across different regions. By addressing these critical areas, substantial progress can be made towards reducing the global burden of burn injuries and improving survival rates in all HDI settings.

## Electronic supplementary material

Below is the link to the electronic supplementary material.

Supplementary Material 1: Figure S 1–7: Distribution of TBSA (darkblue) and age (light blue) by country. Data are expressed as relative frequencies in 5-year and % TBSA increments. AT = Austria, CH = Switzerland, DE = Germany

## Data Availability

The datasets used and/or analyzed during the current study are available from the corresponding author on reasonable request.

## Abbreviations

ABSI: Abbreviated Burn Severity Index
AT: Austria
AU: Australia
BR: Brazil
CH: Switzerland
CI: Confidence Interval
DE: Germany
DGV: German Society of Burn Treatment
GBD: Global Burden of Disease
GDP: Gross Domestic Product
GH: Ghana
HIC: High Income Country
HDI: Human Development Index
IHT: Inhalational Trauma
IN: India
ISO: International Organization for Standardization
LMIC: Low- and Middle-Income Country
mABSI: Modified Abbreviated Burn Severity Index
R: Correlation coefficient r
RW: Rwanda
SD: Standard Deviation
TBSA: Total Body Surface Area
USA: United States of America
WHO: World Health Organization
