# Supplementary figures and images for: Country economic status is strongly associated with burn survival - validation of the (modified) ABSI
Source: Int J Equity Health. 2025 Jan 9;24:5. doi: 10.1186/s12939-024-02353-7 (PMC11715549; doi:10.1186/s12939-024-02353-7)

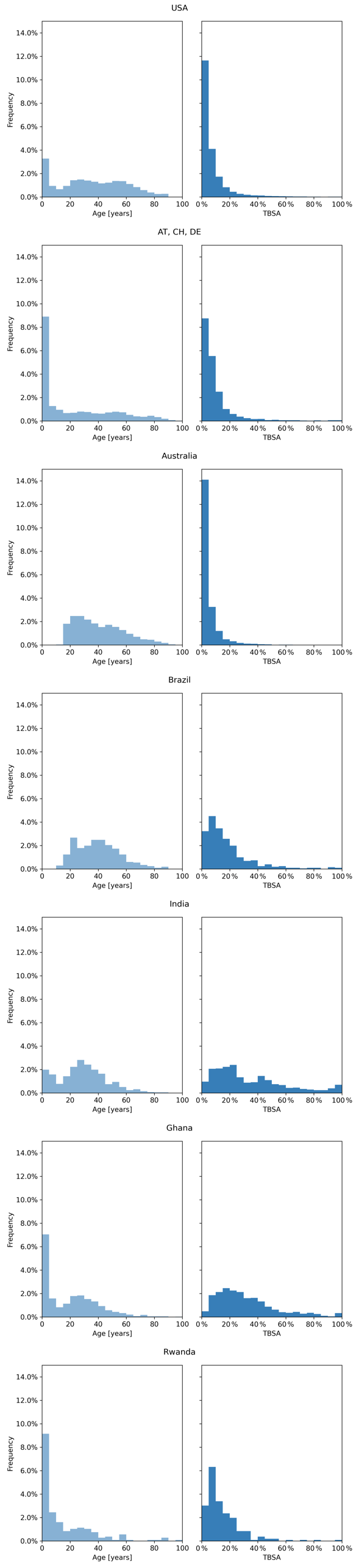

Supplement: Supplementary file 1 — Supplementary Material 1: Figure S 1–7: Distribution of TBSA (darkblue) and age (light blue) by country. Data are expressed as relative frequencies in 5-year and % TBSA increments. AT = Austria, CH = Switzerland, DE = Germany [file 12939_2024_2353_MOESM1_ESM.tiff]
